# Genome-wide host-pathogen analyses reveal genetic interaction points in tuberculosis disease

**DOI:** 10.1038/s41467-023-36282-w

**Published:** 2023-02-01

**Authors:** Jody Phelan, Paula Josefina Gomez-Gonzalez, Nuria Andreu, Yosuke Omae, Licht Toyo-Oka, Hideki Yanai, Reiko Miyahara, Supalert Nedsuwan, Paola Florez de Sessions, Susana Campino, Neneh Sallah, Julian Parkhill, Nat Smittipat, Prasit Palittapongarnpim, Taisei Mushiroda, Michiaki Kubo, Katsushi Tokunaga, Surakameth Mahasirimongkol, Martin L. Hibberd, Taane G. Clark

**Affiliations:** 1 Faculty of Infectious and Tropical Diseases, London School of Hygiene and Tropical Medicine, London, United Kingdom; 2 Department of Human Genetics, Graduate School of Medicine, The University of Tokyo, Tokyo, Japan; 3 Fukujuji Hospital and Research Institute of Tuberculosis, Japan Anti-Tuberculosis Association, Kiyose, Japan; 4 Genome Medical Science Project, National Center for Global Health and Medicine, Tokyo, Japan; 5 Chiangrai Prachanukroh Hospital, Chiangrai, Thailand; 6 Genome Institute of Singapore, One North, Singapore; 7 Department of Veterinary Medicine, University of Cambridge, Cambridge, UK; 8 National Center for Genetic Engineering and Biotechnology, National Science and Technology Development Agency, Pathumthani, Thailand; 9 RIKEN Center for Integrative Medical Sciences, Yokohama, Japan; 10 Medical Genetics Center, Medical Life Sciences Institute, Department of Medical Sciences, Ministry of Public Health, Nonthaburi, Thailand; 11 Faculty of Epidemiology and Population Health, London School of Hygiene & Tropical Medicine, Keppel Street, London, WC1E 7HT United Kingdom

**Keywords:** Genome, Computational biology and bioinformatics

## Abstract

The genetics underlying tuberculosis (TB) pathophysiology are poorly understood. Human genome-wide association studies have failed so far to reveal reproducible susceptibility loci, attributed in part to the influence of the underlying *Mycobacterium tuberculosis* (*Mtb*) bacterial genotype on the outcome of the infection. Several studies have found associations of human genetic polymorphisms with *Mtb* phylo-lineages, but studies analysing genome-genome interactions are needed. By implementing a phylogenetic tree-based *Mtb*-to-human analysis for 714 TB patients from Thailand, we identify eight putative genetic interaction points (P < 5 × 10^−8^) including human loci DAP and RIMS3, both linked to the IFNγ cytokine and host immune system, as well as FSTL5, previously associated with susceptibility to TB. Many of the corresponding *Mtb* markers are lineage specific. The genome-to-genome analysis reveals a complex interactome picture, supports host-pathogen adaptation and co-evolution in TB, and has potential applications to large-scale studies across many TB endemic populations matched for host-pathogen genomic diversity.

## Abstract (web summary)

Few genetic loci have been associated with tuberculosis infection, possibly because of the influence of genetic variation in the pathogen. Here, the authors integrate human and *Mycobacterium tuberculosis* genetics to find genome-genome interactions associated with infection.

## Introduction

Tuberculosis (TB) is a complex disease, caused by *Mycobacterium tuberculosis* (*Mtb*) bacteria, with a wide spectrum of outcomes, probably reflecting differences among human host and pathogen genomes, as well as environmental factors such as immune antigen exposure history. *Mtb* genomic variation, including single nucleotide polymorphisms (SNPs), has helped define *Mtb* lineages and sub-lineages^1,2^. *Mtb* lineages are endemic in different locations around the globe^1,2^, with a phylo-geographical structure, leading to the hypothesis that the strain-types are specifically adapted to people of these global locations and thus different human genetic backgrounds^3,4^. Isolates within lineages 1, 5, and 6 are considered “ancient” strains because they were the first to diverge from the common ancestor, while those in lineages 2 to 4 are called “modern” with some spreading globally and acquiring drug resistance^1,2^. Several studies have reported that lineages may vary in their propensity to transmit and cause severe disease^3,5^; but results are inconsistent and there is considerable inter-strain variation within lineages^1,6^.

Host genetics has the potential to inform about TB disease susceptibility and thus reveal genes important for successful host defence strategies. However, despite the GWAS successes in other diseases^7,8^, this approach has proven difficult for TB^9–12^, with the susceptibility loci identified not replicated in different populations^13,14^. Despite these difficulties, various studies have demonstrated an association between certain alleles of the HLA class II region and pulmonary TB, possibly through reduced presentation of protective *Mtb* antigens to T cells^9,15^. Other GWAS studies have identified loci related to innate immunity^4,13^, known to be important in determining *Mtb* infection and disease outcome^10^. One reason for the difficulty to replicate hits could be the concept of “genetic heterogeneity”, whereby the underlying genetic causes for a trait, in this case susceptibility to TB, may be different across populations. An appealing interpretation of the heterogeneous effect of polymorphisms in different populations implicates the influence of the bacterial genotype on the outcome of the infection for a particular host genotype^16^. Several studies have found associations of some host genetic polymorphisms with particular *Mtb* lineages or strain families^4,17–20^. In particular, associations have been found using targeted or genome-wide host genotyping approaches combined with traditional *Mtb* typing approaches using long sequence polymorphisms or spoligotypes^21–24^. These results suggest that, at least to some extent, TB clinical phenotypes can be explained by the interaction between human and *Mtb* genetic variation. However, these approaches used *Mtb* typing methods that do not fully represent the strain types on a high resolution.

Host-pathogen interaction genomics, using genome-to-genome analytical approaches, has already begun to be used to identify pathogenic mechanisms associated with other diseases, including HIV^25^, hepatitis C virus infection^26^ and Epstein-Barr virus^27^. However, these approaches have not been applied to TB, where genetic studies have predominantly considered human and *Mtb* genomes separately. Here, we sought to reveal insights into human-*Mtb* interaction points using “genome-to-genome” analysis, using a novel approach guided by the *Mtb* phylogenetic structure, thereby controlling for confounding effects of population structure. To this end we integrated the human and *Mtb* genomics data from a well-characterized Thailand cohort (*n* = 714) of TB patients, with an analysis revealing eight putative genetic interaction points (*P* < 5 × 10^−8^), including the involvement of genes related to host immunity.

## Results

### Population structure

**Fig. 1:**
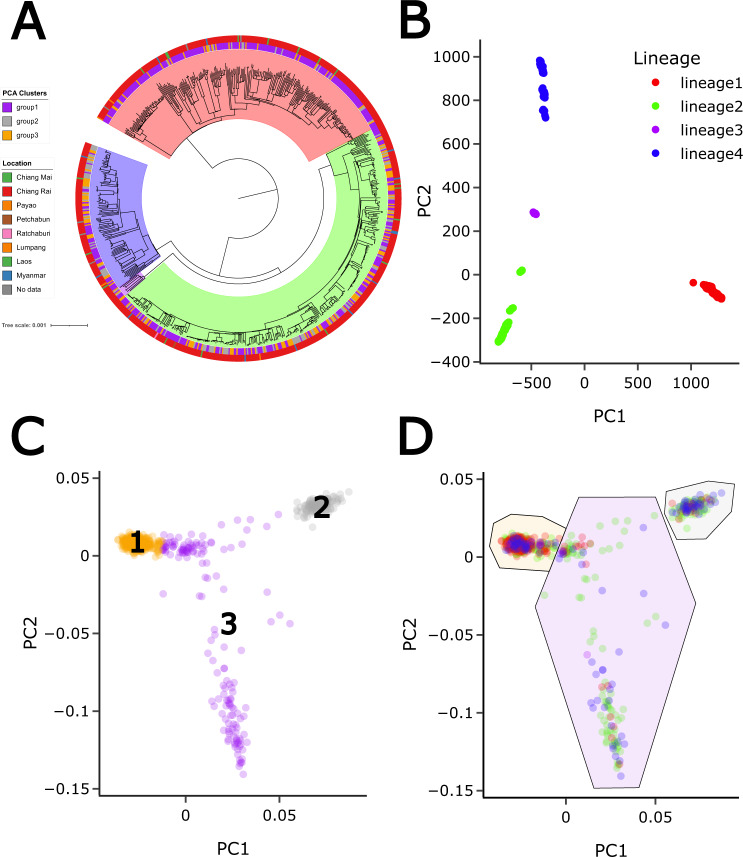
Population structure analysis using *M. tuberculosis* and human genotypes. **A** Phylogenetic tree of the *M. tuberculosis* isolates with location and principal component analysis (PCA) clusters annotated; **B** *M. tuberculosis* population structure based on the first two principal components; **C** human PCA with three main clusters based on k-means clustering shown; (Figure (**B**) with the *M. tuberculosis* lineages superimposed. Lineage 1 strains have a higher prevalence in human cluster 1 (see Supplementary Table 3).

The Thailand cohort (*n* = 714) of TB pulmonary patients were predominantly male (73.7%), all HIV negative and more than 14 years of age (median age: 46 years), with 95% coming from the Chang Rai district in northern Thailand (Supplementary Fig. 1A**;** Supplementary Table 1; Supplementary Table 2). The isolates were predominantly from lineages 1 (35.0%), 2 (47.6%) and 4 (16.2%) (with lineage 3 − 1.1%) (Supplementary Table 3), and 80.4% were predicted to be pan-susceptible across 14 drugs with the remainder having resistance to at least one drug (20.6%). Whole genome sequencing of the *Mtb* isolates identified 56k high-quality genome-wide SNP variants, with the vast majority (92.0%) being rare with minor allele frequencies (MAF) <2%. *Mtb* phylogenetic reconstruction using the 56k SNPs resulted in a tree with 4 major clades, corresponding to the main lineages. Further, subclades defined by long internal branch lengths were also identified (Fig. 1A). Similarly, principal component analysis (PCA) revealed a strong *Mtb* population stratification based on lineages (Fig. 1B; Supplementary Fig. 1B).

Following whole genome SNP chip typing, imputation, and analysis, approximately 7.5 million high-quality SNP variants with MAF >2% were identified across the human samples. Using these SNPs in a PCA approach, the individuals clustered into three groups using the first two components (PCs 1 and 2; Fig. 1C, D). These groups overlap with the East Asian (EAS) group from the 1000 Genomes project (Supplementary Fig. 1C). The three groups maintain their clustering when combined in an EAS sample analysis (Supplementary Fig. 1D**)**, indicating that the diversity measured broadly reflects the genetic diversity in the East Asian region. However, the individuals did not cluster with collection site, suggesting that these clusters are not driven by geography (Supplementary Fig. 1B) and the TB patients are representative more generally of the Thai population. Mapping the *Mtb* lineages onto each the three PCA-based groups revealed an unequal distribution, with lineage 1 being more frequent in PC group 1, and lineage 4 being more frequent in PC groups 2 and 3 (Supplementary Table 3, Chi-Squared *P* = 8.6 × 10^−19^), which suggests nonrandom associations in the human to *Mtb* pairings in our patient population. This could be either due to non-random transmission patterns (e.g., within hospitals) or due to specific human to *Mtb* genetic interaction patterns. The non-random transmission scenario seems unlikely since nearly all isolates are from the relatively small region of Chang Rai and non-familial related individuals (Supplementary Fig. 1; Supplementary Table 2). Recent transmission clusters, defined as *Mtb* isolates with less than 12 SNPs between them^5^, were inferred using calculated SNP distances between all isolate pairs. This analysis revealed six distinct clusters which, except for two small clusters containing only two samples each, contained *Mtb* isolates from all three host groups defined by the PCA (in Fig. 1C) (Supplementary Fig. 2), which suggests mixing and thus supporting the concept of specific affinity of certain human groups to certain *Mtb* lineages.

### Host pathogen interactions

**Fig. 2:**
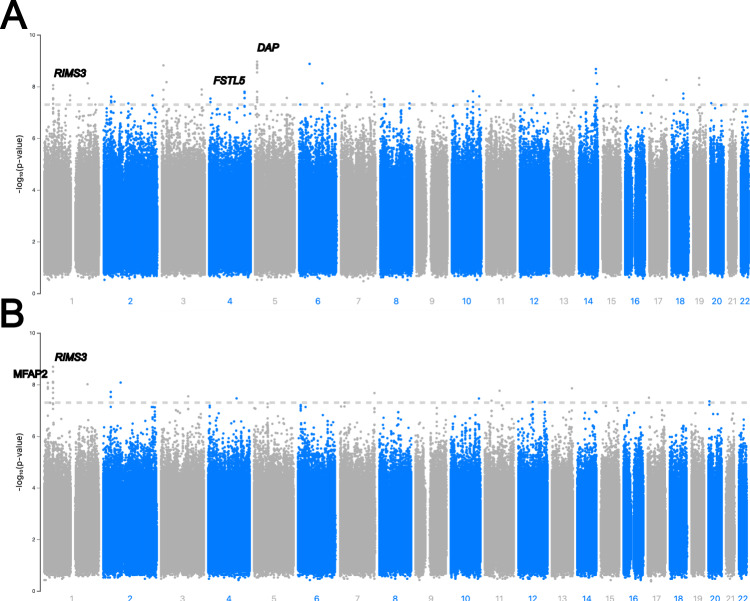
Results from the genome-to-genome comparison of host and pathogen data. A Manhattan plot showing the –log10 (*P* value) for each human variant. Results are plotted by chromosomes with alternating grey and blue colouring. The cut-off (5 × 10^−8^) is shown with the horizontal red line. Results are shown for: (**A**) the whole dataset (*n* = 714), and (**B**) the main host cluster as defined by the principal component analysis (see Fig. 1C) (*n* = 426).

To investigate the genetic basis of the observed differential distribution of strain types among affected individuals, we implemented a human genome-to-*Mtb* genome approach within a GWAS regression framework using all host and pathogen pairs (see METHODS). This method allowed us to test for associations between internal nodes on the *Mtb* phylogenetic tree (144 with a minimum clade proportion > 2%) and human variants (~7.4 million with MAF > 2%), adjusting for the confounding effects of both *Mtb* and host population structure (see METHODS). The analysis revealed associations involving 105 human SNPs (in 44 loci) and 30 *Mtb* phylogenetic clades (*P* < 5 × 10^−8^; Fig. 2; Table 1). These included findings in or close to host genes RIMS3 (chromosome 1; 14 SNPs), FSTL5 (chr. 4; 10 SNPs), DAP (chr. 5; 18 SNPs), CSGALNACT1 (chr. 8; 3 SNPs), as well as gene deserts on chromosomes 2 (6 SNPs), 3 (3 SNPs) and 14 (4 SNPs) (Supplementary Fig. 3). The RIMS3 gene encodes a protein that is involved in a diverse range of biological functions, including pulmonary-related, and the regulation of synaptic membrane exocytosis with some evidence of regulation by IFNγ, a cytokine linked to the immune system^28^. Several SNPs in a region immediately downstream of this gene were associated with a *Mtb* clade within lineage 1, containing lineage 1.1.1 strains (Supplementary Fig. 4). Both FSTL5 and DAP proteins were associated with a clade containing fifteen lineage 2.2.1 (Beijing) strains. SNPs in the FSTL5 gene, which codes for a protein involved in calcium binding, has been associated with susceptibility to TB in an ancestry-adjusted association analysis^29^. DAP codes for a protein involved in mediation of cell death induced by IFNγ. CSGALNACT1 is an enzyme involved in the biosynthesis of alternative forms of glycosaminoglycans, namely chondroitin sulfate, linked to B cell activity, as well as multiple sclerosis progression^30^.

**Table 1:** Genome-to-genome association results

Host Chr.	Host Region	No. SNPs^a^	SNP^b^	*P* value	Odds ratio	Host Locus	Host Locus Annotation	*Mtb* Clade lineage	Analysis^c^
5	10712199–10758562	18	rs267951	1.41 × 10^−9^	40.52	DAP	Intronic	2.2.1	All
14	97134528–97150790	4	rs74875032	2.11 × 10^−9^	21.47	Intergenic	–	4.4.2	All
1	17303792–17310019	5	rs529617685	8.57 × 10^−9^	129.69	MFAP2	Intronic	2.2.1.1	Main
4	162602209–162620104	10	rs142600697	1.59 × 10^−8^	42.49	FSTL5	Intronic	2.2.1	All
2	35360834–35367230	6	rs1118438	2.47 × 10^−8^	22.78	Intergenic	–	1.1.3	All, Main
1	41067739–41074312	14	rs558237	2.86 × 10^−8^	3.61	RIMS3	Downstream	1.1	All, Main
3	8308620–8310990	3	rs59441182	3.12 × 10^−8^	19.79	Intergenic	–	4.4.2	All
8	19413249–19418028	3	rs4563899	4.84 × 10^−8^	29.27	CSGALNACT	Intronic	2.2.1	All

The minimum P-value per gene and the associated odds ratio and lineage of the *M. tuberculosis* variant (*Mtb*).

^a^Number of SNPs with *P* < 5×10^−8^;

^b^the SNP with the strongest association (minimum *P* value);

^c^Analyses were performed using all paired samples (*n* = 714) and the main cluster only (*n* = 426) as determined using the first two principal components (see Fig. 1C).

It is noteworthy that previous work has suggested an association between variants on the human genome with both susceptibility to infection and to specific strain types^9,10,12,13,21,22,24,31^. The statistical significance of these previously reported hits in the current study was checked by extracting the minimum *p* value in a 20kbp region centred around the reported SNP (Supplementary Table 4). The most significant p-values were found on an intergenic region on chromosome 18 (*P* = 5.41 × 10^−7^; rs4331426) described by Thye et al.^12^, and in the HLA region on chromosome 6 (*P* = 4.39 × 10^−6^; rs9272785) reported by Sveinbjornsson et al.^9^. Although none of these sequence variants in our analysis reach the significance cut-off, a potential marginal peak was observed around HLA-DQB1 (*P* = 4.92 × 10^−7^) (Supplementary Fig. 4). HLA class II sequence variants have been linked to susceptibility to TB infection^9^. The most significant peak across the HLA region was observed close to HLA-E (*P* = 1.68 × 10^−7^), but this did not reach genome-wide significance (Supplementary Fig. 4).

To reveal if the putative interactions were human cluster specific, host-pathogen pairs from just the main PC group (cluster 1, *n* = 426, Fig. 1C) were analysed, leading to associations between 40 human SNPs across 16 different loci with 15 *Mtb* phylogenetic clades (Table 1**;** Supplementary Fig. 3). These included associations with human SNPs overlapping or near to the RIMS3 gene (16 SNPs), MFAP2 (5 SNPs) and a gene desert on chromosome 2 (6 SNPs). MFAP2 is a glycoprotein which is a component of elastin-associated microfibrils, with SNPs in this gene associated with chronic obstructive pulmonary disease and lung function^32^. As indicated above, the association hits were spread across different lineages and subclades of the *Mtb* phylogeny indicating there may be several different interaction points between the host and pathogen genomes (Supplementary Fig. 5). For the top human association SNP hits identified (Table 1), there are differences in the variability in the allele frequencies across Thailand and 1000 Genome project populations, and the linked lineage prevalence in comparable geographical regions from a 32k *Mtb* dataset^1^ (Supplementary Table 5). Across these SNPs, there is some evidence of correlations between major human allele frequency and *Mtb* lineage in regional populations (Spearman correlations mean: −0.151, range: −0.673 − 0.322), reinforcing a highly complex dynamic between human host and pathogen across geography, and the need to consider both organisms to understand TB biology, pathophysiology, and epidemiology.

To ascertain whether any of the association hits have been the subject of recent positive selective pressure across the three human clusters, a selection analysis was performed using the between group XP-EHH metric. This analysis revealed that a region surrounding rs59441182 (chr. 3) had elevated XP-EHH values (Supplementary Fig. 6) when comparing human cluster 2 to others (XP-EHH > 3; *P* < 3 × 10^−3^), consistent with the interaction effect not being driven by an analysis of the main cluster. The other hits showed no association. As a benchmark, the same analysis was also run on the MHC region, where selection signals are expected, and revealed high XP-EHH values (>3) across all three pairwise cluster comparisons (Supplementary Fig. 6).

## Discussion

Genome-to-genome studies have been proposed to elucidate the complex interplay between host and pathogen genetics. One of the first studies reported associations between human genetic variants and 48 HIV-1 amino acid variants in 1071 HIV-infected patients, where all associated host SNPs mapped to the HLA class I region and none of the viral amino acids mapped to known sites of major antiretroviral drug resistance mutations^25^. Similarly, a study of Epstein-Barr virus found significant associations between human and viral sequence variation, involving three polymorphic regions in the human genome, including SNPs on chromosome 7, and a variant in the BRLF1 gene of the virus^27^. Host-pathogen co-evolution has also been proposed as an interaction mechanism for TB, however, the diversity found among *Mtb* differs from the viral setting. Importantly, excluding drug resistance mutations, most of the variation seen in *Mtb* isolates is either very rare or is lineage specific. Due to the long history of human-*Mtb* co-evolution in TB, thought to span thousands of years^3^, different clades have arisen with up to 920 unique defining mutations^1^. This evolutionary process makes pinpointing of specific variations in the pathogen genome difficult as they are effectively in perfect linkage disequilibrium. While there have been several studies reporting the association between different strain types and host genotypes, a study using pathogen genomic data to perform a high-resolution association analysis informed by phylogenetic clades has been lacking.

By testing associations between all possible combinations of human SNP variation and *Mtb* clades, we have highlighted significant associated human variants, including those in RIMS3, MFAP2 and DAP, which have links to host immunity. This suggests that susceptibility to TB follows a complicated pattern with many host factors involved, coupled with the diversity within the *Mtb* pathogen, which itself has surprisingly large impacts on function^33^. Human populations could differ in their susceptibility to different lineages of *Mtb*, thereby supporting the TB host-pathogen co-evolution hypothesis^3^. Our data suggests that if this is confirmed, then robust human SNP-*Mtb* lineage interactions seen in one geographical location, such as identified in this study, would rarely be observed in other regions of the world, highlighting the difficulties in achieving replicated findings using traditional case-control and GWAS studies. One of the limitations of the current study is the fact that non-genetic factors, including socio and epidemiological, could have potential to limit the spread of certain strain types. For example, non-mixing between different ethnic groups through socio or geographic factors could limit the potential transmission between groups, leading to an enrichment of some strain types with host genetic groups, rather than it being driven by host genotypes. The relative importance of the putative genetic interactions identified must now be investigated through follow-up (e.g., replication) studies in different populations. Our analysis suggests that large-scale numbers across paired samples matched for host-pathogen genomic diversity would facilitate replication. The resulting insights from such investigations will be instrumental for designing and informing treatment and vaccine-design decisions for TB that may be more specific to infecting strains or host genetics, thereby providing much-needed control measures to assist disease elimination.

## Methods

### Study population

The 714 TB cases were recruited from hospitals in Chiang Rai, Lampang and Bangkok provinces in Thailand (TB incidence 181/100,000 population) between 2010 and 2012. These included a minority of travellers from neighbouring Laos and Myanmar. The patients had no previous history of TB disease, were primarily male (73.9%), and aged > 14 years. The TB diagnosis was confirmed by microscopy and culture, and all individuals are HIV seronegative (see Supplementary table 1).

### Ethics

The project was approved by the Ethical Committees of Chiangrai Prachanukroh Hospital, Chiangrai and the Thai Ministry of Public Health. Informed consent was obtained from all participants and/or their legal guardians. All methods were performed in accordance with the relevant guidelines and regulations.

### Genetic data

Human genotypes for the Thai TB cases (*n* = 714) were generated on Illumina Human610-Quad and Illumina HumanOmniExpressExome-8 v1.2 BeadChips, complemented by imputation of >8.4 million genomic sites using Beagle4.1 software^34^ and a 1000 Genomes reference panel^35^. Human leukocyte antigen (HLA) protein alleles were imputed using SNP2HLA software (v1.0.3) and a pan-Asian reference^36^. SNPs were removed if there was: (i) deviation in genotypic frequencies from Hardy-Weinberg equilibrium (HWE) as assessed using a chi-square test (*P* < 0.00001); (ii) high genotype call missingness (>10%); (iii) low minor allele frequency (<5%); or (iv) low imputation quality (allelic *R*^2^ < 0.7). The final number of SNPs was 5,948,940. The population structure was explored using principal component (PC) analysis (PCA) inferred from pairwise SNP genotype differences between individuals using Plink2 (v2.00a3.7LM) software (settings --pca). This analysis led to three clear human PC clusters. To check the extent of this variability compared to global genetic variation, the data was merged with the 1000 Genome project set and PCA was performed on the combined dataset as well as with only the East Asian (EAS) populations.

*M. tuberculosis* (*Mtb*) sequence data was generated at the Sanger Institute using an Illumina HiSeq2000 machine. The raw sequencing data were aligned to the H37Rv reference genome (Genbank accession number: NC_000962.3) using the BWA-MEM algorithm (v0.7.17-r1188)^37^. SAMtools/BCFtools^38^ software was used to call SNPs and small indels using default options. Alleles were additionally called across the whole genome (including SNP sites) using a coverage-based approach. A missing call was assigned if the total depth of coverage at a site did not reach a minimum of 20 reads or none of the four nucleotides accounted for at least 75% of the total coverage. Samples or SNP sites having an excess of 10% missing genotype calls were removed. This quality control step was implemented to remove samples with low quality genotype calls due to poor depth of coverage or mixed infections. The final discovery dataset included 720 Thai isolates and ~59k genome-wide SNPs. SNPs were combined within a multifasta format file and used as input to iq-tree software for phylogenetic reconstruction (v2.1.4, -m GTR + G + ASC). Custom scripts were used to traverse the tree and create input files for Plink2 software to run the association analysis detailed below (https://github.com/jodyphelan/host-pathogen, 10.5281/zenodo.7528265). Lineages and drug resistance were predicted using the TB-Profiler tool (v4.2.0)^39^. The *Mtb* SNP analytical pipeline is described in greater detail elsewhere^40^. To look for evidence of recent transmission between samples, the number of SNP differences between all pairs of samples was calculated. A cut-off of 12 SNPs was used to infer transmission^41^, and putative transmission clusters were visualised using a customised javascript script.

### Statistical analysis

To uncover effects between lineages and the human PC group overall, a χ^2^ test was applied. The genome-to-genome analysis was performed using logistic regression with the *Mtb* phylogenetic clade (binary variable: in or not in clade) as the outcome and human genotypes as predictors. *Mtb* clades were included in the analysis if their nodes represented > 2% of the total number of samples. A separate logistic model was fitted for each human SNP genotypic effect (additive, heterozygous, dominant, recessive, general), and the minimum p-value across the tests for each *Mtb* clades was retained. The two main human PCs were included in the model to adjust for population structure in the analysis of “all” Thailand samples. A sensitivity analysis was performed on the main association hits, and assessed whether highly significant *P *values were robust to models that included 0, 2, 5 or 10 PCs. All association hits were robust to applying these different numbers of PCs. As the PCA revealed three distinct clusters with the main cluster representing ~60% of the data, the analysis steps were repeated using only samples from this cluster without PC adjustment in the model. This analysis is referred to in the text as the “main cluster” analysis as opposed to the “all” Thai sample analysis. *P* values were used to rank association hits. Given the complexity of establishing significance thresholds in host-pathogen genomics, a cut-off of *P* < 5 × 10^−8^ was used as a guide to establish and present the most significant hits from the analysis. This significance threshold is similar to a recent human genome-to-pathogen genome study^26^. Regional association plots were generated using *locuszoom*^42^ using the minimum *P* value across the different genotypic models and tested clades for each human variant, to produce a single Manhattan plot for the analysis of all data and for the main cluster analysis. For comparisons, *Mtb* (sub-)lineage data was available for 32k globally sourced isolates^1^. Selection analysis was performed on human genotypes using a between-population XP-EHH approach implemented in selscan software (v2.0.0; --xpehh), and attempted to establish if the association hits were in genetic regions with evidence of recent positive selection. Three separate analyses were conducted in pairwise comparisons of the human clusters identified using the host PCA. Annotated maps were generated using the maps library package within the R statistical software tool.

### Reporting summary

Further information on research design is available in the Nature Portfolio Reporting Summary linked to this article.

## Supplementary information

Supplementary Information

Reporting Summary

## Data Availability

The pathogen raw sequencing data generated in this study have been deposited in the ENA sequence read archive database under accession code PRJEB7056. The reference genome used to map the pathogen data was sourced from the NCBI nuccore database under the accession code NC_000962.3. The 1000 genome reference panel VCF files were sourced from the Beagle4.1 software website and are available to download at https://bochet.gcc.biostat.washington.edu/beagle/1000_Genomes_phase3_v5a/. Summary statistics for the genome-to-genome analysis are available for download at https://github.com/jodyphelan/host-pathogen.

## Code availability

Analysis scripts are available for download at https://github.com/jodyphelan/host-pathogen.
