# Supplementary material for: Genome-wide host-pathogen analyses reveal genetic interaction points in tuberculosis disease
Source: Nat Commun. 2023 Feb 1;14:549. doi: 10.1038/s41467-023-36282-w (PMC9892022; doi:10.1038/s41467-023-36282-w)
Supplement: Supplementary file 1 — Supplementary Information [file 41467_2023_36282_MOESM1_ESM.pdf]

Supplementary Information

Genome-wide host-pathogen analyses reveal genetic interaction points in tuberculosis disease

Jody E. Phelan[1], Paula Josefina Gomez-Gonzalez[1], Nuria Andreu[1], Yosuke Omae[2], Licht Toyo-Oka[2], Hideki Yanai[3], Reiko Miyahara[4], Supalert Nedsuwan[5], Paola Florez de Sessions[6], Susana Campino[1], Neneh Sallah[1], Julian Parkhill[7], Nat Smittipat[8], Prasit Palittapongarnpim [8], Taisei Mushiroda[9], Michiaki Kubo[9], Katsushi Tokunaga[2], Surakameth Mahasirimongkol[10], Martin L. Hibberd[1,*], Taane G. Clark[1,11,*]

[1] Faculty of Infectious and Tropical Diseases, London School of Hygiene and Tropical Medicine, London, United Kingdom

[2] Department of Human Genetics, Graduate School of Medicine, The University of Tokyo, Tokyo, Japan.

[3] Fukujuji Hospital and Research Institute of Tuberculosis, Japan Anti-Tuberculosis Association, Kiyose, Japan.

[4] Genome Medical Science Project, National Center for Global Health and Medicine, Tokyo, Japan.

[5] Chiangrai Prachanukroh Hospital, Chiangrai, Thailand

[6] Genomics Institute Singapore, Singapore

[7] Department of Veterinary Medicine, University of Cambridge, Cambridge, UK.

[8] National Center for Genetic Engineering and Biotechnology, National Science and Technology Development Agency, Pathumthani, Thailand

[9] RIKEN Center for Integrative Medical Sciences, Yokohama, Japan

[10] Medical Genetics Center, Medical Life Sciences Institute, Department of Medical Sciences, Ministry of Public Health, Nonthaburi, Thailand.

[11] Faculty of Epidemiology and Population Health, London School of Hygiene & Tropical Medicine, Keppel Street, London, WC1E 7HT, United Kingdom

* joint corresponding authors

Professor Martin Hibberd (martin.hibberd@lshtm.ac.uk) and

Professor Taane G. Clark (taane.clark@lshtm.ac.uk)

Supplementary figure 1

Population analysis

A) Map labelled with the number of samples from geographical regions represented in this study; B) A principal component analysis (PCA) plot with all collection sites outside Chiang Rai (main site) highlighted; C) and D) Population structure analysis of Thailand TB cases (n=714) using genome-wide genotypes led to genetic differences with other Asian populations (n=504) within the clusters (C1-C3 from Figure 1). PCA of the genotypes from the Thailand TB hosts and: C) All 1000 Genomes populations groups in regions (European; East Asian; American; South Asian; African); D) East Asian and Thailand controls

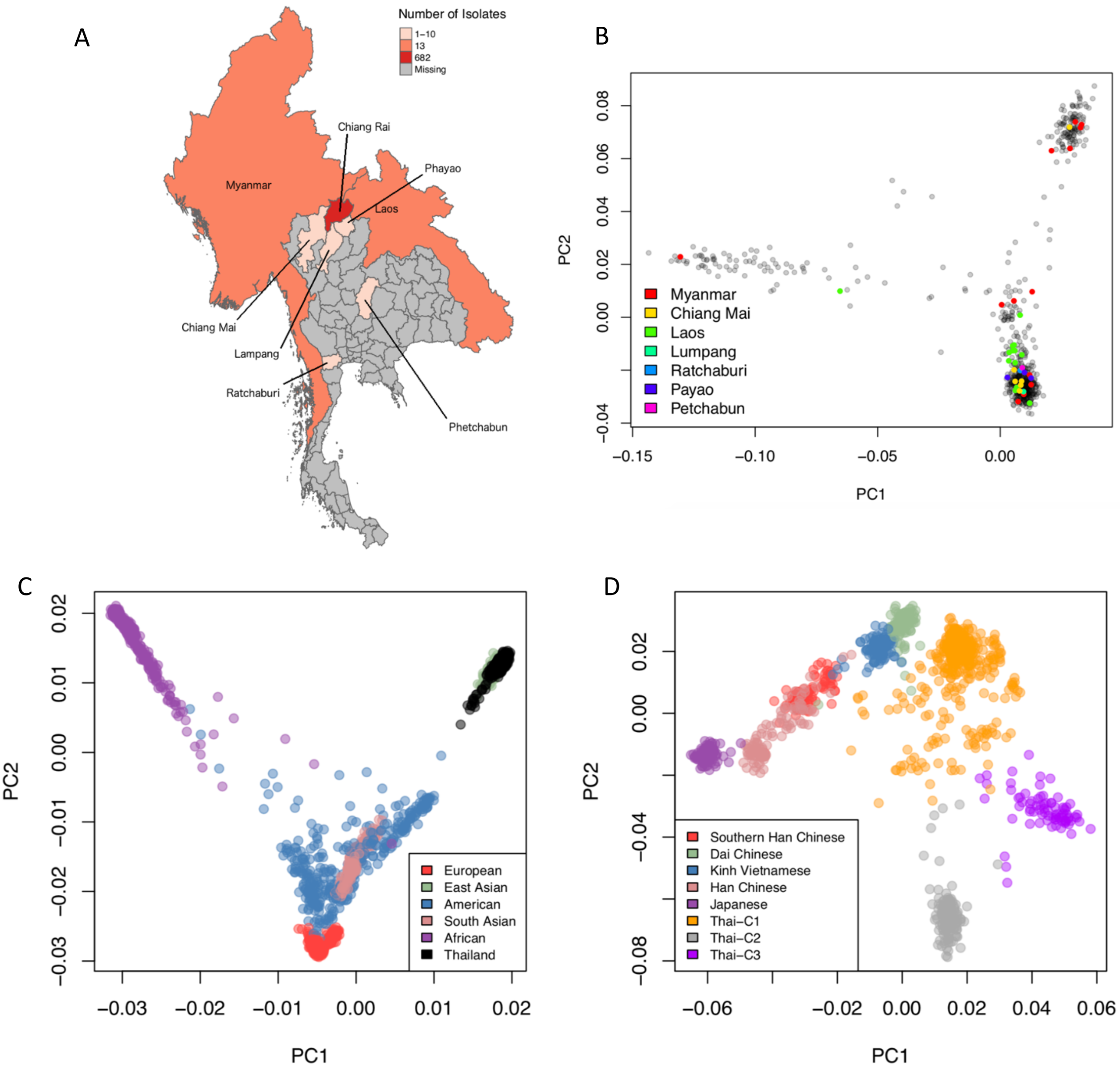

Supplementary figure 2

Transmission analysis showing the six transmission clusters across the *M. tuberculosis* isolates. The lines in each clade represent links between the *M. tuberculosis* isolates that have <12 SNPs difference. The colours represent the three human groups as per the PCA clusters shown in Figure 1c.

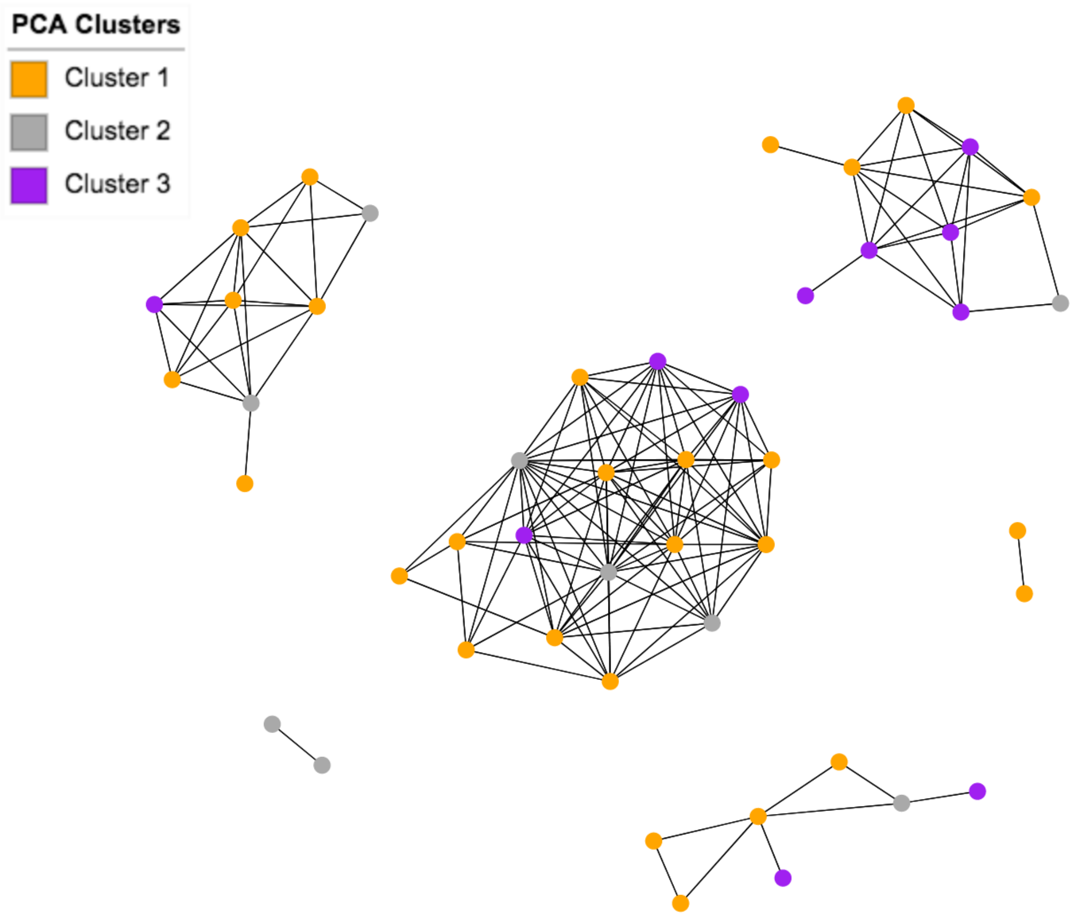

Supplementary figure 3

Host genetic regions identified as having putative interactions with the *M. tuberculosis* genome. Regional association plots showing the P-values of the identified SNPs plotted against their physical position in the chromosome (Genome build hg19). Recombination rate as estimated from the 1,000 genomes Asian population is plotted in light blue. Purple diamond: SNP with strongest evidence for association. The color of each SNP indicates linkage of disequilibrium (LD) with the SNP with the strongest association, based on pairwise r^2 values from 1000 Genomes project data (http://www.internationalgenome.org). Gene annotations from the hg19 genome browser are shown.

(a) *DAP* (all samples)

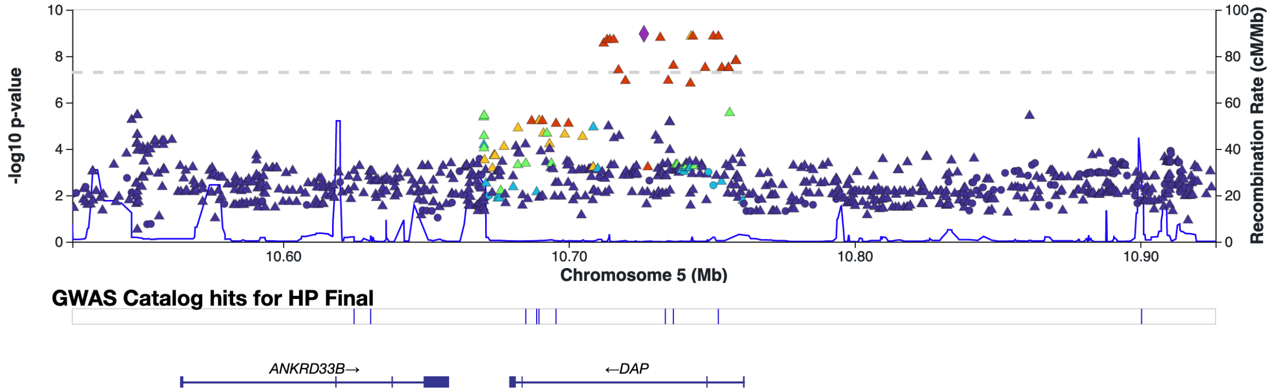

(b) Intergenic chromosome 14 (all samples)

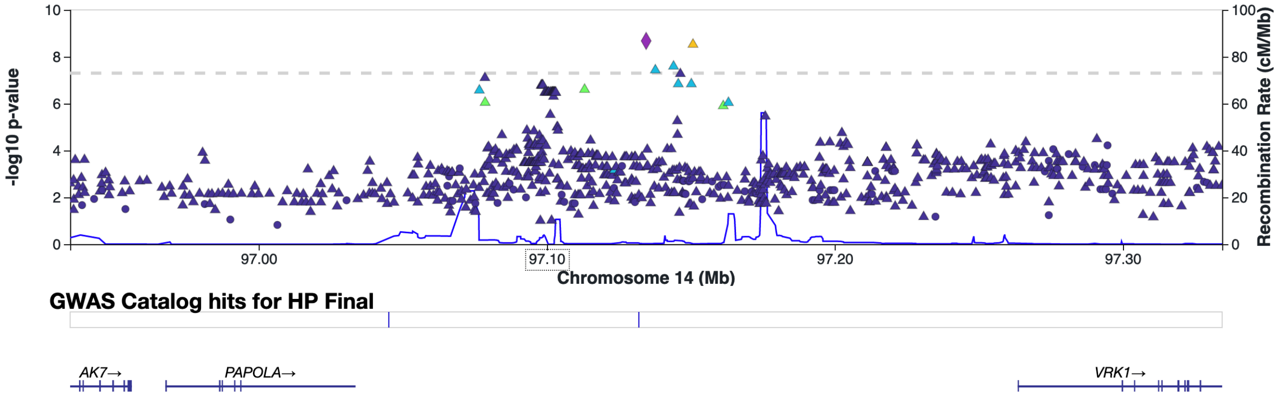

(c) *MFAP2* (main cluster)

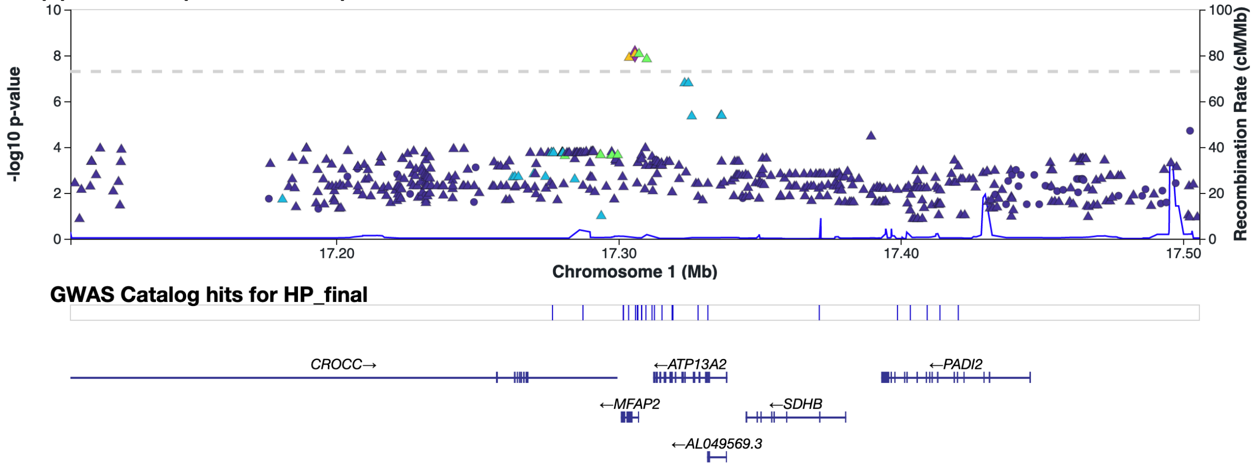

(d) *FSTL5* (all samples)

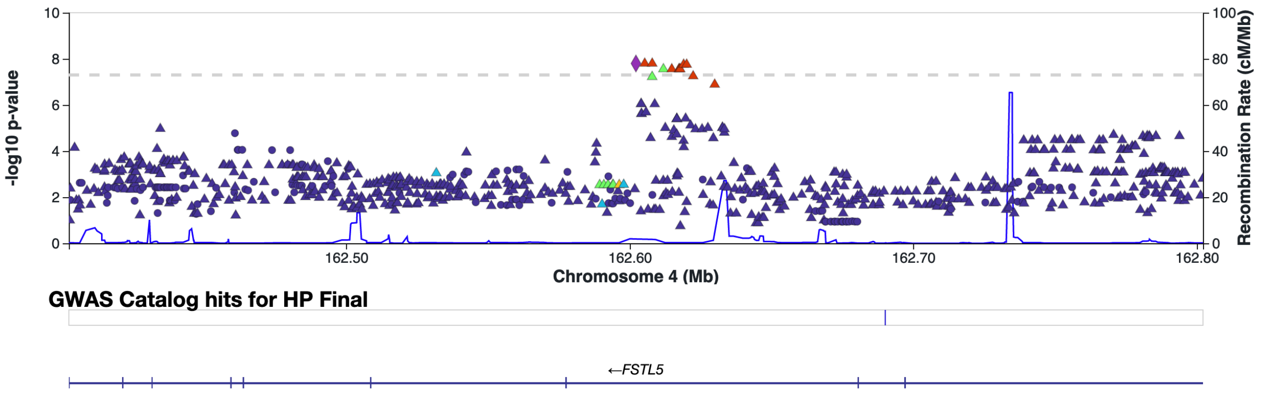

(e) Intergenic chromosome 2 (main cluster and all samples)

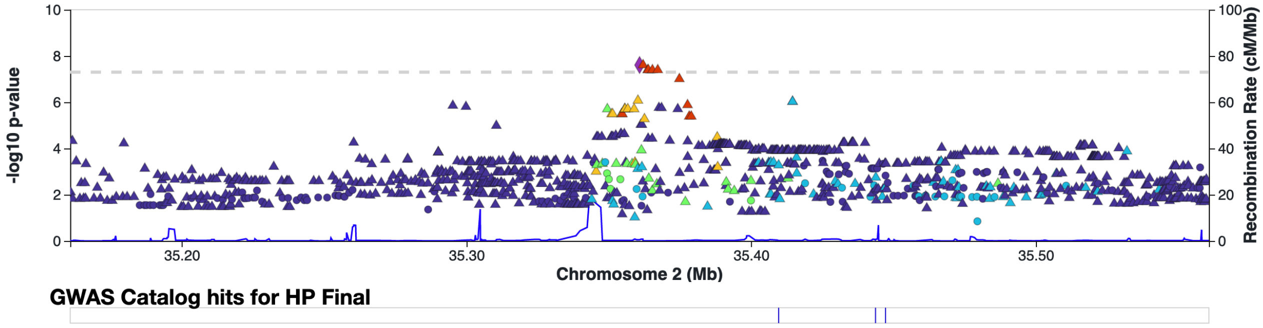

(f) *RIMS3* (main cluster)

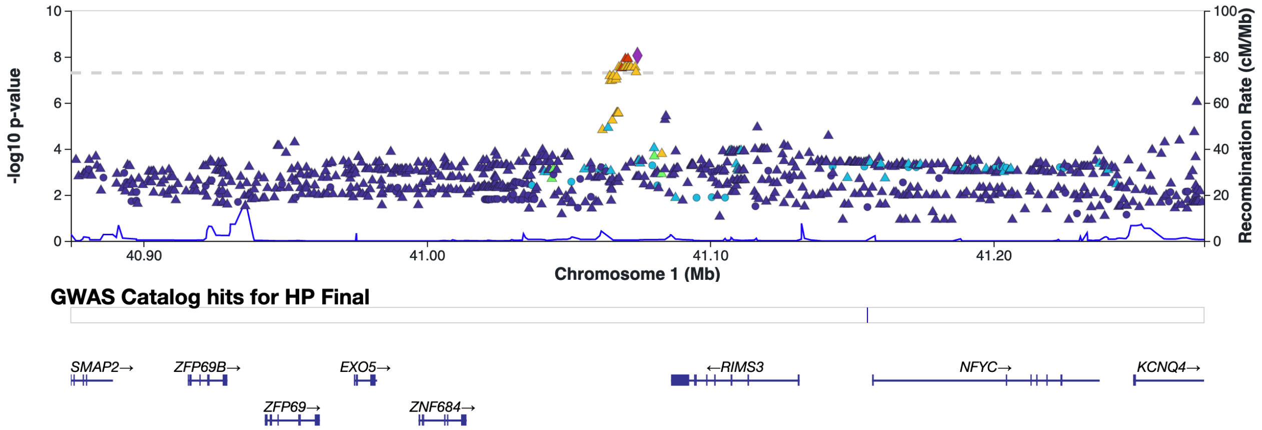

(g) Intergenic chromosome 3 (all samples)

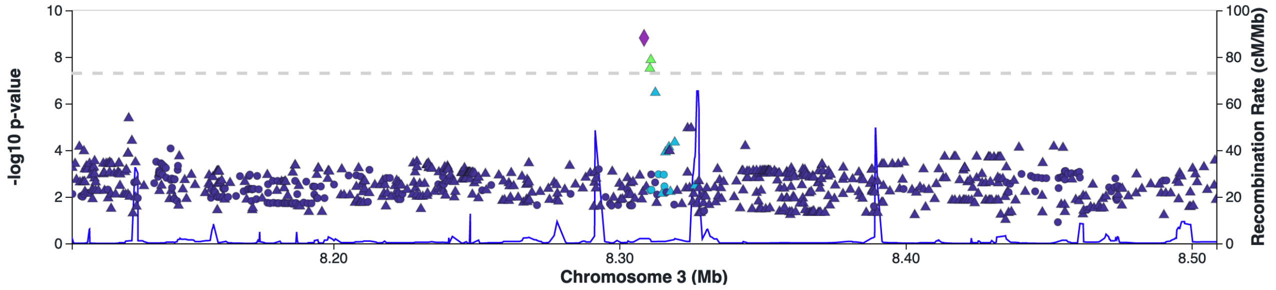

(h) *CSGALNACT* (all samples)

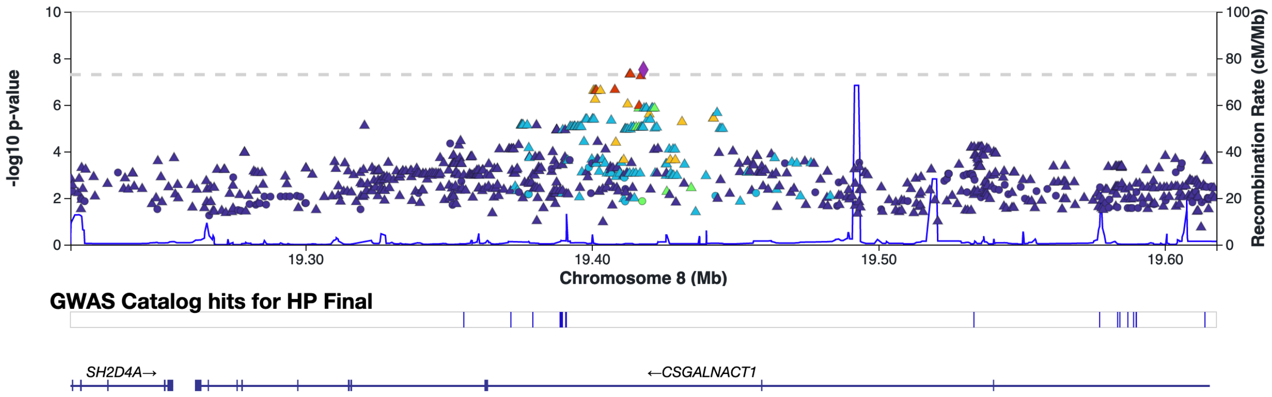

10
8
6
4
2
0
-log10 p-value
100
80
60
40
20
0
Recombination Rate (cM/Mb)
19.30
19.40
19.50
19.60
Chromosome 8 (Mb)
GWAS Catalog hits for HP Final
SH2D4A→
←CSGALNACT1

Supplementary figure 4
Manhattan plots showing peaks around (a) HLA-DQB1 and (b) HLA-E

(a)

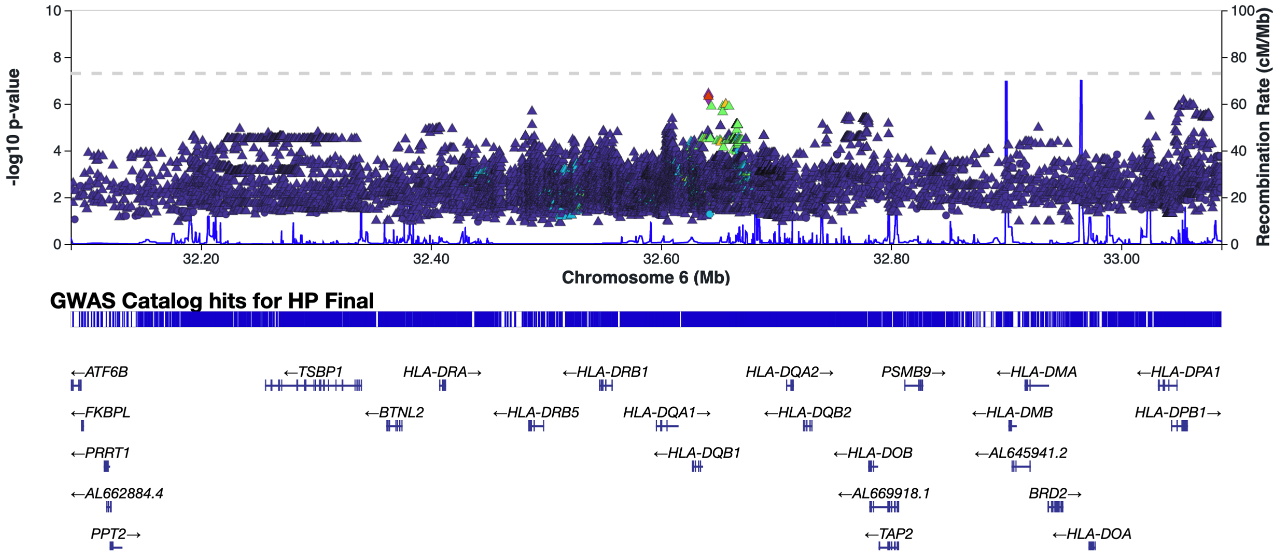

(b)

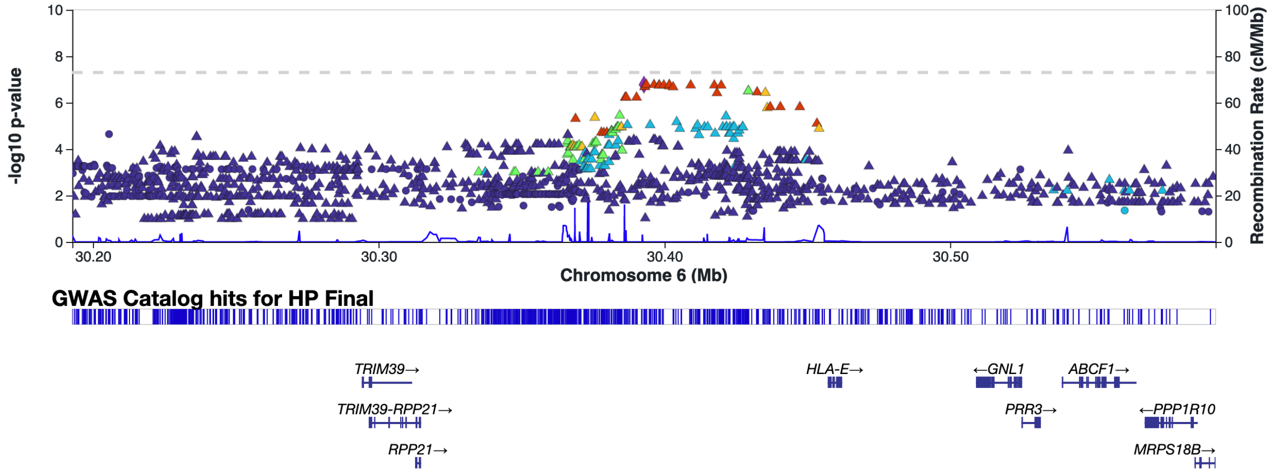

Supplementary figure 5
A phylogenetic tree for the Thailand *M. tuberculosis* (n=714) with the top host genome-to-genome association hits (rs numbers) and associated nodes highlighted (black bands).

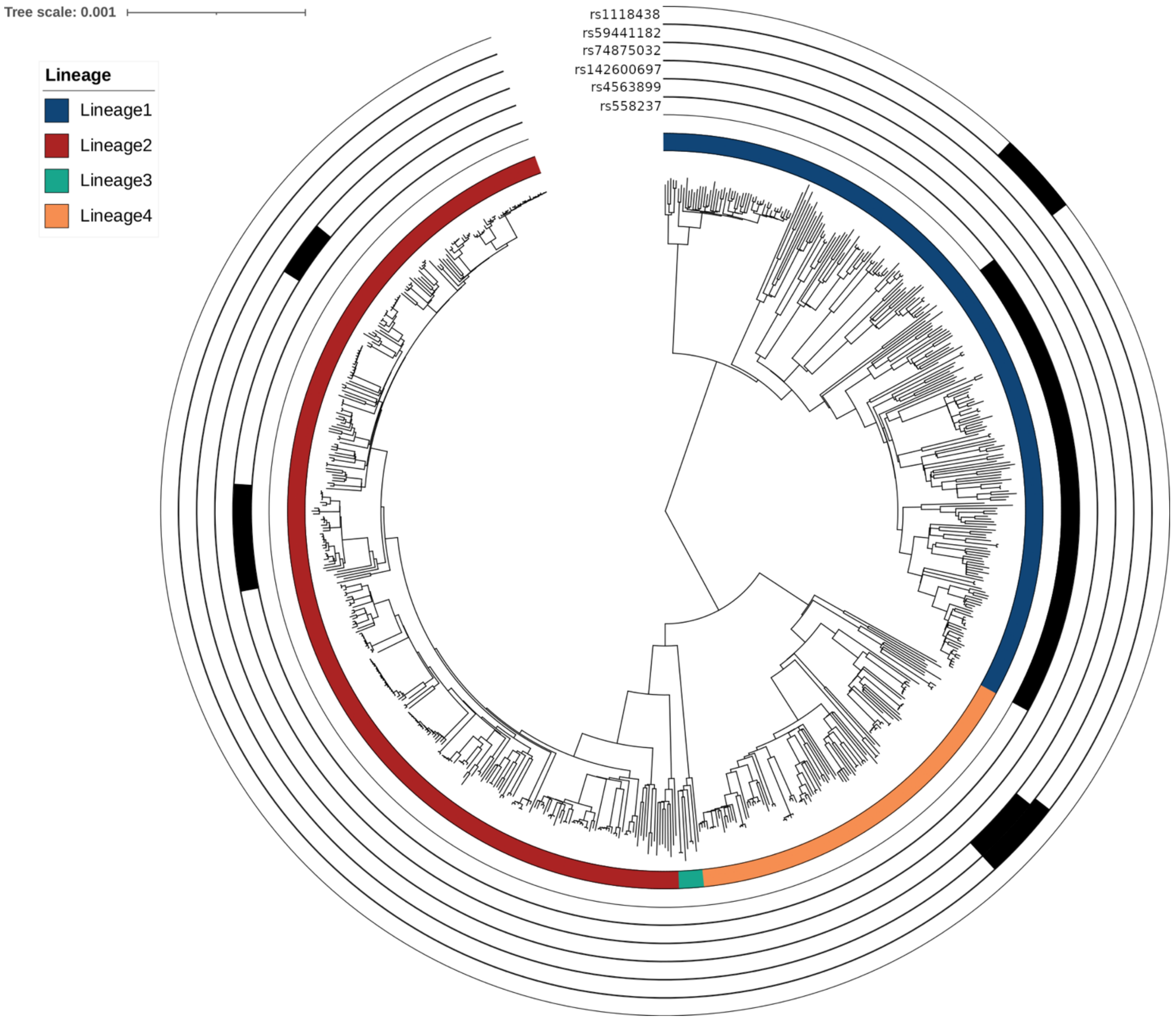

Supplementary Figure 6

Recent positive selection using the XP-EHH metric, comparing the 3 human PCA clusters (see Figure 1c) pairwise. A to C are for a 20 kbp region surrounding SNP rs59441182. D to F are for the MHC region (5 Mbp). A and D compare human clusters 1 and 2; B and E compare clusters 2 and 3; C and F compare clusters 1 and 3. Absolute values of XP-EHH > 3 (dashed red line; P=0.0027) and 4 (solid red line, P=6.3x10^{-5}) are indicative of strong selective pressure.

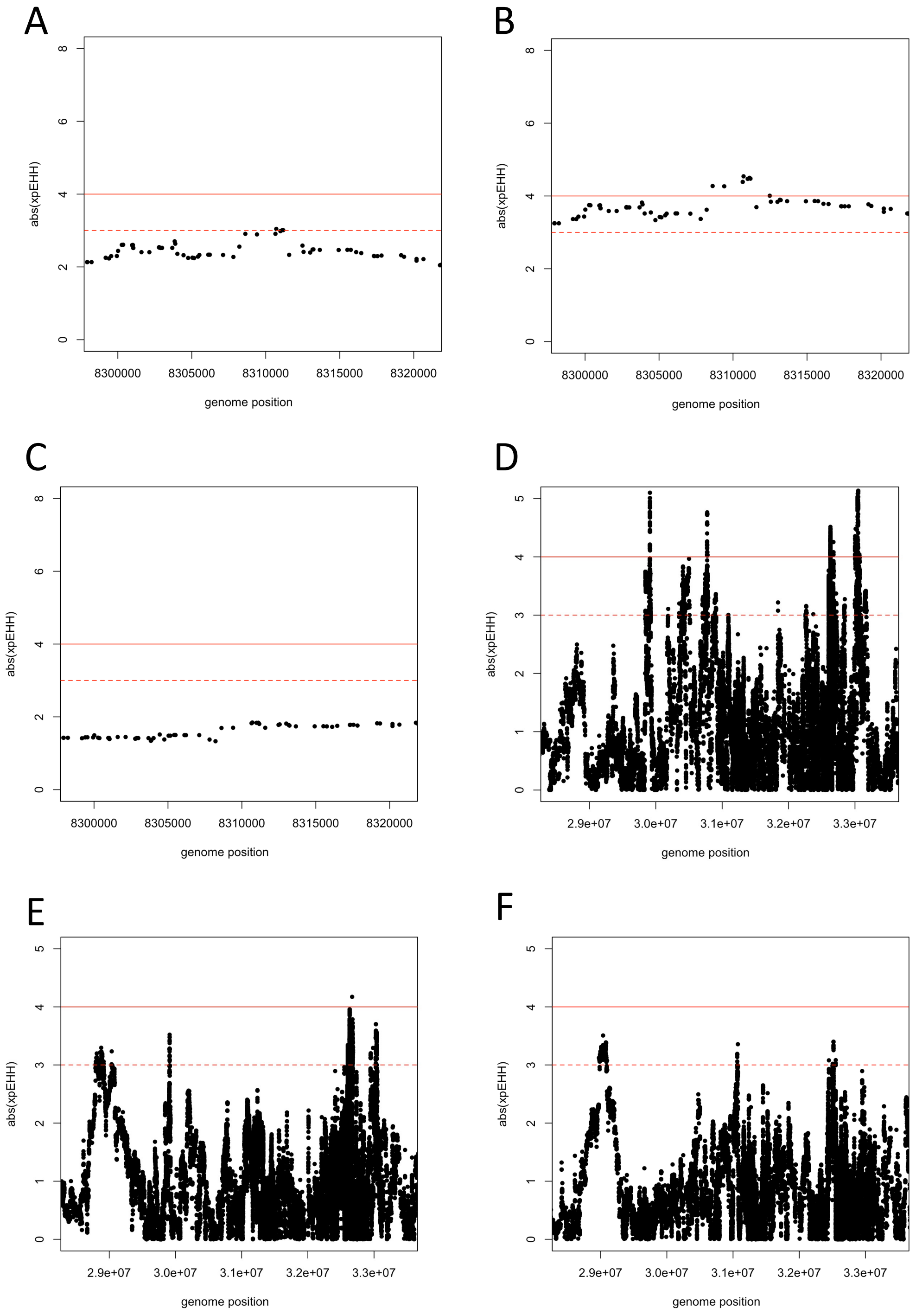

Supplementary table 1
Baseline characteristics for the 714 TB cases

Characteristics	N	%
Age (years)		
15-17	21	2.9
18-35	195	27.3
36-50	224	31.4
>50	274	38.4
Sex		
Female	188	26.3
Male	526	73.7
HIV status		
negative	714	100
Location		
Chiang Rai	677	94.8
Chiang Mai	6	0.8
Payao	3	0.4
Petchabun	1	0.1
Lumpang	1	0.1
Ratchaburi	1	0.1
Myanmar	13	1.8
Laos	12	1.8

Supplementary table 2
Geographic source of the *M. tuberculosis* isolates and their lineages

Region	N (%)	Lineage 1	Lineage 2	Lineage 3	Lineage 4
Chiang Rai	677 (94.8)	238 (35.2)	321 (47.4)	8 (1.2)	110 (16.2)
Chiang Mai	6 (0.8)	1 (16.7)	4 (66.7)	0 (0)	1 (16.7)
Payao	3 (0.4)	2 (66.7)	1 (33.3)	0 (0)	0 (0)
Petchabun	1 (0.1)	0 (0)	1 (100)	0 (0)	0 (0)
Lumpang	1 (0.1)	0 (0)	0 (0)	0 (0)	1 (100)
Ratchaburi	1 (0.1)	0 (0)	1 (100)	0 (0)	0 (0)
Myanmar*	13 (1.8)	2 (15.4)	9 (69.2)	0 (0)	2 (15.4)
Laos*	12 (1.8)	7 (61.5)	3 (23.1)	0 (0)	2 (15.4)
Total (%)	714 (100)	250 (35.3)	340 (47.4)	8 (1.1)	116 (16.1)

* Travellers from non-Thailand regions

Supplementary table 3
The distribution of *M. tuberculosis* lineages within each human PCA-based cluster group.

M. tuberculosis Lineage*	Human Cluster 1	Human Cluster 2	Human Cluster 3	Total
1	208 (48.8%)	15 (12.0%)	27 (16.6.6%)	250 (35.0%)
2	170 (39.9%)	71 (56.8%)	99 (60.7%)	340 (47.6%)
3	4 (0.9%)	2 (1.6%)	2 (1.2%)	8 (1.1%)
4	44 (10.3%)	37 (29.6%)	35 (21.5%)	116 (16.2%)
Total	426	125	163	714

* there is evidence of a non-random association between lineage and cluster (Chi-Sq. $P = 8.6 \times 10^{-19}$)

Supplementary table 4
Candidate regions identified in previous studies

Gene	Identifier [Ref]	Location*	Main cluster min P-value	All samples min P-value
NRAMP1	rs17235409[24]	2: 219259732	1.01×10^{-3}	7.23×10^{-4}
NRAMP1	rs3731865[24]	2: 219250003	2.53×10^{-5}	5.82×10^{-5}
MARCO	rs2278589[22]	2: 119728876	5.10×10^{-5}	1.06×10^{-3}
MARCO	rs6751745[22]	2: 119741004	2.67×10^{-5}	2.87×10^{-4}
PPIAP22	rs114945555[21]	21: 20187488	7.41×10^{-5}	4.93×10^{-5}
EREG	rs7675690[31]	4: 75248651	5.73×10^{-4}	2.33×10^{-4}
ASAP1	rs4733781[10]	8: 131296767	3.87×10^{-4}	3.51×10^{-4}
ASAP1	rs10956514[10]	8: 131252758	2.04×10^{-4}	3.51×10^{-4}
-	rs4331426[12]	18: 20190795	5.41×10^{-7}	2.11×10^{-6}
JAG1	rs2273061[13]	20: 10639543	1.50×10^{-4}	4.50×10^{-4}
DYNLR	rs4461087[13]	16: 79991016	9.06×10^{-4}	1.68×10^{-3}
EBF1	rs10515787[13]	5: 158475211	1.48×10^{-4}	1.37×10^{-3}
TMEFF2	rs10497744[13]	2: 194490896	4.97×10^{-3}	1.74×10^{-3}
TMEFF2	rs1020941[13]	2: 194475270	5.69×10^{-4}	1.74×10^{-3}
CCL17	rs188872[13]	16: 57452165	5.40×10^{-4}	1.35×10^{-4}
HAUS6	rs10245298[13]	7: 53656669	1.78×10^{-4}	2.46×10^{-4}
PENK	rs6985962[13]	8: 57434777	3.47×10^{-5}	2.56×10^{-5}
TXNDC4	rs1418267[13]	9: 102806574	1.92×10^{-4}	2.49×10^{-4}
MHC	rs557011[9]	6:32587013	1.55×10^{-4}	1.08×10^{-4}
MHC	rs9271378[9]	6:32587300	1.55×10^{-4}	1.08×10^{-4}
MHC	rs9272785[9]	6:32610401	4.39×10^{-6}	6.18×10^{-6}

* human chromosome: Starting position

Supplementary table 5

Major allele frequencies for interacting human SNPs identifed within Thailand (see Table 1) and compared to continental populations for 1000 genomes data, with frequencies of *M. tuberculosis* (*Mtb*) lineage in comparable populations from a 32k dataset.

Human SNP and *Mtb* lineage pairs	THA	EAS	AFR	AME	SAS	EUR
rs267951	0.881	0.874 (0.844-0.900)	0.775 (0.737-0.818)	0.511 (0.477-0.535)	0.701 (0.563-0.784)	0.433 (0.401-0.491)
Lineage 2.2.1	0.446	0.237 (0.119-0.472)	0.189 (0.000-0.773)	0.043 (0.000-0.090)	0.049 (0.000-0.100)	0.140 (0.081-0.328)
rs74875032	0.976	0.988 (0.970-1.000)	1.000 (1.000-1.000)	0.986 (0.976-0.992)	0.958 (0.948-0.971)	0.999 (0.995-1.000)
Lineage 4.4.2	0.027	0.007 (0.000-0.021)	0.000 (0.000-0.000)	0.000 (0.000-0.001)	0.000 (0.000-0.000)	0.001 (0.000-0.002)
rs529617685	0.169	0.149 (0.121-0.171)	0.493 (0.434-0.532)	0.111 (0.076-0.149)	0.288 (0.238-0.377)	0.187 (0.162-0.224)
Lineage 2.2.1	0.026	0.034 (0.005-0.091)	0.000 (0.000-0.000)	0.005 (0.000-0.013)	0.009 (0.000-0.018)	0.007 (0.000-0.012)
rs142600697	0.887	0.843 (0.814-0.876)	0.930 (0.900-0.949)	0.923 (0.918-0.930)	0.816 (0.711-0.872)	0.953 (0.921-0.972)
Lineage 2.2.1	0.446	0.237 (0.119-0.472)	0.189 (0.000-0.773)	0.043 (0.000-0.090)	0.049 (0.000-0.100)	0.140 (0.081-0.328)
rs1118438	0.168	0.206 (0.195-0.220)	0.642 (0.593-0.682)	0.585 (0.435-0.734)	0.578 (0.544-0.617)	0.774 (0.727-0.797)
Lineage 1.1.3	0.038	0.003 (0.000-0.005)	0.000 (0.000-0.000)	0.000 (0.000-0.000)	0.041 (0.000-0.167)	0.006 (0.000-0.015)
rs558237	0.594	0.539 (0.500-0.575)	0.577 (0.535-0.625)	0.551 (0.532-0.562)	0.666 (0.587-0.733)	0.499 (0.430-0.621)
Lineage 1.1.3	0.038	0.003 (0.000-0.005)	0.000 (0.000-0.000)	0.000 (0.000-0.000)	0.041 (0.000-0.167)	0.006 (0.000-0.015)
rs59441182	0.796	0.689 (0.657-0.707)	0.584 (0.566-0.593)	0.689 (0.660-0.712)	0.541 (0.466-0.588)	0.617 (0.545-0.681)
Lineage 4.4.2	0.027	0.007 (0.000-0.021)	0.000 (0.000-0.000)	0.000 (0.000-0.001)	0.000 (0.000-0.000)	0.001 (0.000-0.002)
rs4563899	0.854	0.859 (0.838-0.876)	0.509 (0.435-0.580)	0.805 (0.761-0.836)	0.662 (0.607-0.755)	0.792 (0.769-0.833)
Lineage 2.2.1	0.446	0.237 (0.119-0.472)	0.189 (0.000-0.773)	0.043 (0.000-0.090)	0.049 (0.000-0.100)	0.140 (0.081-0.328)

THA = Thailand, EAS = East Asia, AFR = Africa, AME = Americas, SAS = South Asia, EUR = Europe
