# Supplementary material for: Genome-wide host-pathogen analyses reveal genetic interaction points in tuberculosis disease
Source: Nat Commun. 2023 Feb 1;14:549. doi: 10.1038/s41467-023-36282-w (PMC9892022; doi:10.1038/s41467-023-36282-w)
Supplement: Supplementary file 2 — Reporting Summary [file 41467_2023_36282_MOESM2_ESM.pdf]

nature portfolio

Corresponding author(s): Taane Clark

Last updated by author(s): 2023/01/13

Reporting Summary

Nature Portfolio wishes to improve the reproducibility of the work that we publish. This form provides structure for consistency and transparency in reporting. For further information on Nature Portfolio policies, see our Editorial Policies and the Editorial Policy Checklist.

Statistics

For all statistical analyses, confirm that the following items are present in the figure legend, table legend, main text, or Methods section.

n/a	Confirmed	
☐	☒	The exact sample size (*n*) for each experimental group/condition, given as a discrete number and unit of measurement
☐	☒	A statement on whether measurements were taken from distinct samples or whether the same sample was measured repeatedly
☐	☒	The statistical test(s) used AND whether they are one- or two-sided<br>*Only common tests should be described solely by name; describe more complex techniques in the Methods section.*
☐	☒	A description of all covariates tested
☐	☒	A description of any assumptions or corrections, such as tests of normality and adjustment for multiple comparisons
☐	☒	A full description of the statistical parameters including central tendency (e.g. means) or other basic estimates (e.g. regression coefficient) AND variation (e.g. standard deviation) or associated estimates of uncertainty (e.g. confidence intervals)
☐	☒	For null hypothesis testing, the test statistic (e.g. *F*, *t*, *r*) with confidence intervals, effect sizes, degrees of freedom and *P* value noted<br>*Give P values as exact values whenever suitable.*
☒	☐	For Bayesian analysis, information on the choice of priors and Markov chain Monte Carlo settings
☒	☐	For hierarchical and complex designs, identification of the appropriate level for tests and full reporting of outcomes
☒	☐	Estimates of effect sizes (e.g. Cohen's *d*, Pearson's *r*), indicating how they were calculated

Our web collection on statistics for biologists contains articles on many of the points above.

Software and code

Policy information about availability of computer code

Data collection	No software was used for the data collection
Data analysis	Genomic imputation was performed using beagle (v4.1). HLA imputation was performed using SNP2HLA (v1.0.3). Plink2 (v2.0) was used to perform all analysis of genotype data including QC, PCA and statistical tests. Analysis of bacterial sequence data was performed using bwa (v0.7.17) for mapping and samtools/bcftools (v1.12) for variant calling. Phylogenetic reconstruction was performed using iq-tree (v2.1.4). Custom scripts were used to generate input files for plink from the phylogenetic tree and to perform all downstream analysis (https://github.com/jodyphelan/host-pathogen, DOI:10.5281/zenodo.7528265). Selscal (v2.0.0) was used to scan for selective sweeps.

For manuscripts utilizing custom algorithms or software that are central to the research but not yet described in published literature, software must be made available to editors and reviewers. We strongly encourage code deposition in a community repository (e.g. GitHub). See the Nature Portfolio guidelines for submitting code & software for further information.

Data

Policy information about availability of data

All manuscripts must include a data availability statement. This statement should provide the following information, where applicable:

- Accession codes, unique identifiers, or web links for publicly available datasets
- A description of any restrictions on data availability
- For clinical datasets or third party data, please ensure that the statement adheres to our policy

The pathogen raw sequencing data generated in this study have been deposited in the ENA sequence read archive database under accession code PRJEB7056. The reference genome used to map the pathogen data was sourced from the NCBI nuccore database under the accession code NC_000962.3. The 1000 genome reference panel VCF files were sourced from the beagle4.1 website and are available to download at https://bochet.gcc.biostat.washington.edu/beagle/1000_Genomes_phase3_v5a/.

Human research participants

Policy information about studies involving human research participants and Sex and Gender in Research.

Reporting on sex and gender	Sex was collected during recruitment, but did not influence study design. The study cohort was primarily male (N=526 - 73.9%). No sex-specific analysis was performed as this is not thought to play a role in host-pathogen interactions. In addition, the patients recruited had ages between 17 and 40 years old, and were HIV-negative, with no previous history of TB.
Population characteristics	The patients had no previous history of TB disease, were primarily aged > 14 years (97.1%). The TB diagnosis was confirmed by microscopy and culture, and all individuals are HIV seronegative.
Recruitment	TB patients were recruited from the Chiangrai Prachanukroh Hospital, Chiangrai. All patients attending the hospital between 2010 and 2012 were invited to participate to genomic investigations, and the majority provided consent (>80%), with minimal bias and unlikely to substantially impact the results.
Ethics oversight	The project was approved by the Ethical Committees of Chiangrai Prachanukroh Hospital, Chiangrai and the Thai Ministry of Public Health.

Note that full information on the approval of the study protocol must also be provided in the manuscript.

Field-specific reporting

Please select the one below that is the best fit for your research. If you are not sure, read the appropriate sections before making your selection.

☒ Life sciences ☐ Behavioural & social sciences ☐ Ecological, evolutionary & environmental sciences

For a reference copy of the document with all sections, see nature.com/documents/nr-reporting-summary-flat.pdf

Life sciences study design

All studies must disclose on these points even when the disclosure is negative.

Sample size	The study consisted of a convenience sample. The sample size (714) was deemed to be sufficient to detect a moderate effect.
Data exclusions	No data was excluded.
Replication	Cohorts are currently being recruited to replicate the results of our discovery study.
Randomization	This is a cohort study, not a clinical trial, and randomisation was not applicable.
Blinding	This was a cohort study and blinding was not applicable.

Reporting for specific materials, systems and methods

We require information from authors about some types of materials, experimental systems and methods used in many studies. Here, indicate whether each material, system or method listed is relevant to your study. If you are not sure if a list item applies to your research, read the appropriate section before selecting a response.

Materials & experimental systems

n/a	Involved in the study
☒	☐ Antibodies
☒	☐ Eukaryotic cell lines
☒	☐ Palaeontology and archaeology
☒	☐ Animals and other organisms
☒	☐ Clinical data
☒	☐ Dual use research of concern

Methods

n/a	Involved in the study
☒	☐ ChIP-seq
☒	☐ Flow cytometry
☒	☐ MRI-based neuroimaging
